# Log-transformed variance from individual growth curves as a potential indicator of resilience in Nile tilapia *Oreochromis niloticus*

**DOI:** 10.1038/s41598-025-91353-w

**Published:** 2026-03-02

**Authors:** Muhammad Hunaina Fariduddin Aththar, Samuel Bekele Mengistu, John A.H. Benzie, Hans Komen, John W.M. Bastiaansen

**Affiliations:** 1 Animal Breeding and Genomics, Wageningen University & Research, Wageningen, the Netherlands; 2 Research Center for Applied Zoology, National Research and Innovation Agency (BRIN), Cibinong, Indonesia; 3 WorldFish, Kudu Road Plot 39, Kabulonga, Lusaka, Zambia; 4 School of Animal and Range Sciences, College of Agriculture, Hawassa University, Awassa, Ethiopia; 5 WorldFish, Jalan Batu Maung, , Batu Maung, Bayan Lepas, Penang, Malaysia; 6 School of Biological Earth and Environmental Sciences, University College Cork, Cork, Ireland

**Keywords:** Variance of deviation of individual growth, Resilience, Nile tilapia, Heritability, Genetic improvement, Genetics, Animal breeding

## Abstract

The ability of the animal to cope with environmental changes may be measured by log-transformed variance of deviations from expected weights (LnVar). We calculate LnVar by fitting the expected individual growth curve based on longitudinal weights (LnVar_ind_) of Nile tilapia that were grown in either an aerated or a non-aerated freshwater pond. We estimated genetic parameters for LnVar_ind_ in Nile tilapia, the genetic correlation between LnVar_ind_ and growth and the genetic correlation for LnVar_ind_ between aerated and non-aerated pond. The heritability estimate for LnVar_ind_ (0.28) in the non-aerated pond was higher than in aerated pond (0.06). In the aerated pond, genetic correlations of LnVar_ind_ were − 0.44 ± 0.23 with daily growth coefficient (DGC) and − 0.45 ± 0.24 with harvest weight (W_5_). In the non-aerated pond, genetic correlations with DGC and W_5_ were − 0.68 ± 0.12 and − 0.52 ± 0.17, respectively. These values suggest that selection for fish with high growth rate will reduce LnVar_ind_. However, genetic correlation of LnVar_ind_ between aerated and non-aerated pond was 0.50, suggesting that genetic improvement in the aerated environment will reduce LnVar_ind_ in the non-aerated environment. Therefore, incorporating records from relatives in non-aerated pond is beneficial for breeding programs targeting this environment.

## Introduction

All animals, including fish, respond to environment disturbances with changes in behaviour and increased levels of stress hormones, such as cortisol and adrenalin^1–3^. Stress, either acute or chronic, affects feed intake, digestion, and ultimately growth^4–6^. However, the magnitude of these responses can vary significantly between individuals. This has led to the idea that individual deviations in growth over time could be used as an indicator for sensitivity to stressful conditions. Resilience is then defined as the ability of the animal to cope with environmental perturbations or to rapidly return to the condition it had before exposure to a disturbance^7–10^. Resilience is also seen as the ability to have consistent performance throughout time. Animals that show consistent performance are expected to be less affected by environmental perturbations than animals with less consistent production. Therefore, resilience indicators can be based on observed production variations even though the causes of these variations are unknown^9,10^. Resilience, expressed as the consistency in growth, can be measured from the deviations of actual weight from the expected weight in longitudinal measurements^7,11^. Several indicators to measure resilience from the deviation of actual weight have been proposed^10^. Of these, $\:LnVar$ is the most promising based on its moderate heritability and ease of calculation from longitudinal records^12–14^.

Ideally, the reference used to calculate individual deviations would be as close as possible to the trajectory that a fish would have realized in the absence of disturbances. Therefore, it is important to estimate a reference or expected performance that is independent of environmental conditions. A previous study by Mengistu, et al.^14^ measured fish response to the disturbance using $\:LnVar$, calculated based on individual deviations from the mean weight of the fish cohort ($\:{LnVar}_{coh}$). However, the changes in the mean weight of the fish cohort are also dependent on environmental conditions across various time points. Consequently, the response of individual fish to the disturbance, calculated based on the mean weight of the fish cohort, is relative to the response of the group or cohort to environmental changes. An alternative approach is to fit an expected growth curve from longitudinal measurement of fish weights. Growth curves can be fitted using weight and age in calendar days or temperature days^15^. Feeding levels also affect growth rate but animals in commercial production are typically fed to satiation.

In aquaculture and fisheries, nonlinear functions to model the age–weight relation have been intensively used to describe the growth curve of different aquatic species, including the Gompertz function, von Bertalanffy growth function and Schnute function^15^. The application of nonlinear models for fitting growth curves was also helpful in describing the growth in Nile tilapia^16^. Most of these growth models are based on the metabolic growth model, assuming that growth depends on weight exponent of 2/3[17, 18]. von Bertalanffy reasoned that the area of surfaces involved in anabolism is proportional to a linear dimension squared and that the weight related to catabolism is proportional to a linear dimension cubed^17,18^. The weight exponent of fish can be estimated from weight data using nonlinear regression. Mayer, et al.^19^ and Janssen, et al.^20^ estimated a weight exponent of ~ 2/3 in Seabream.

In this study, we apply non-linear regression to fit individual growth curves using longitudinal weight measurements of tilapia grown in Malaysia (described in detail in Mengistu, et al.^14^). Tilapia were grown in an aerated and non-aerated freshwater pond and weight was measured at 5-time points during the grow-out period. The individual growth curves were then used to calculate $\:LnVar$ ($\:{LnVar}_{ind}$). We hypothesize that $\:LnVar$ measures growth resilience and therefore should be expressed more in the non-aerated environment due to high fluctuations in dissolved oxygen levels. The objectives of this study were: (1) to estimate genetic parameters of $\:{LnVar}_{ind}\:$in Nile tilapia (*Oreochromis niloticus*), (2) to estimate the genetic correlation between $\:{LnVar}_{ind}\:$and growth to explore the effects of selection for growth rate on $\:{LnVar}_{ind}$ and (3) to estimate the genetic correlation for $\:{LnVar}_{ind}$ between aerated and non-aerated pond.

## Results

**Fig. 1:**
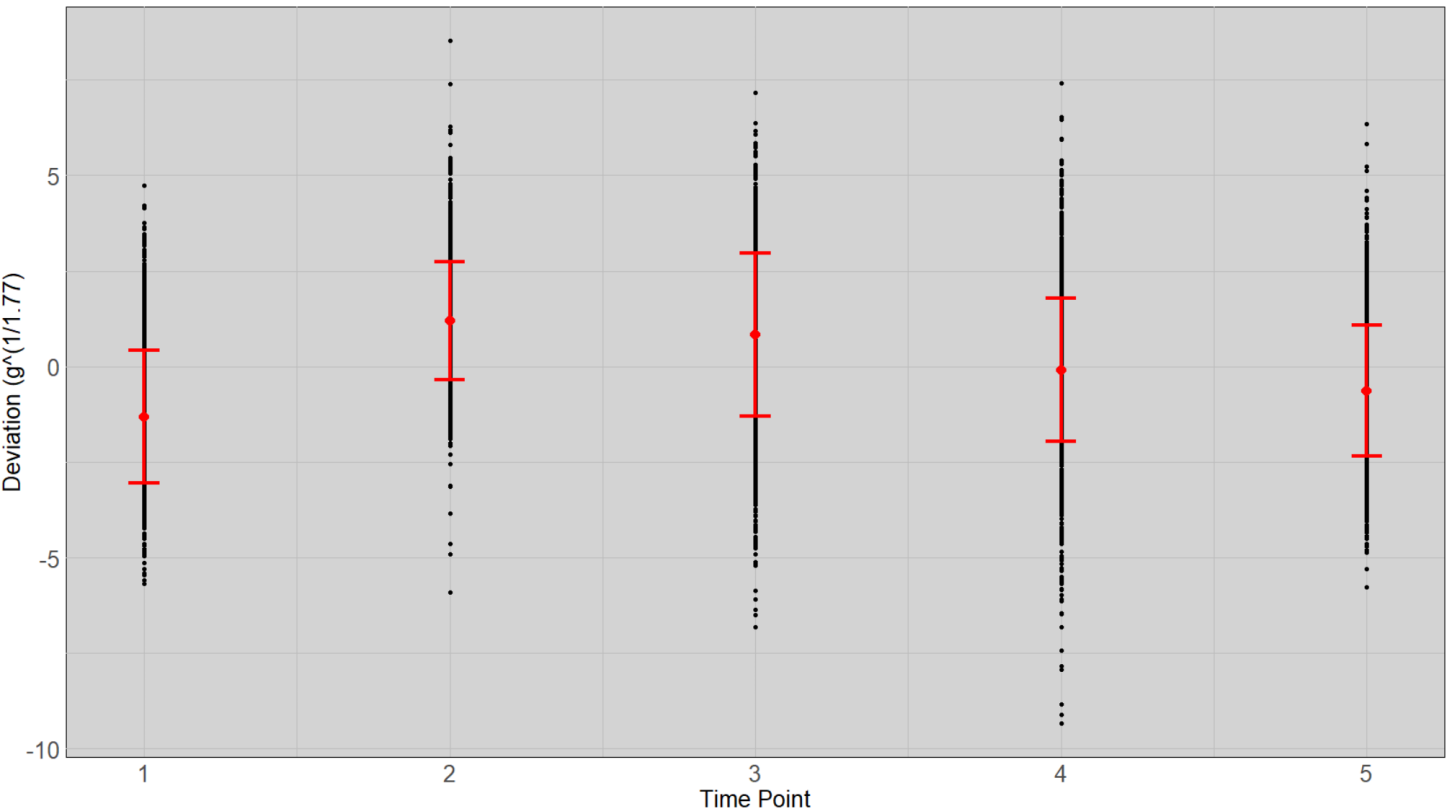
Mean and standard deviation of the weight deviation from expected individual weight (gram) with non-linear regression in $$\:\raisebox{1ex}{$1$}\!\left/\:\!\raisebox{-1ex}{$f$}\right.$$ scale at stocking ($$\:{T}_{1}$$), three interval time points ($$\:{T}_{2-4}$$: 55, 104 and 167 days) and at harvest ($$\:{T}_{5}$$: 217 days) for all fish. The weight of each fish is plotted as a dot with the standard error limits shown by two short horizontal lines and the mean is located at the mid-point between these.

**Fig. 2:**
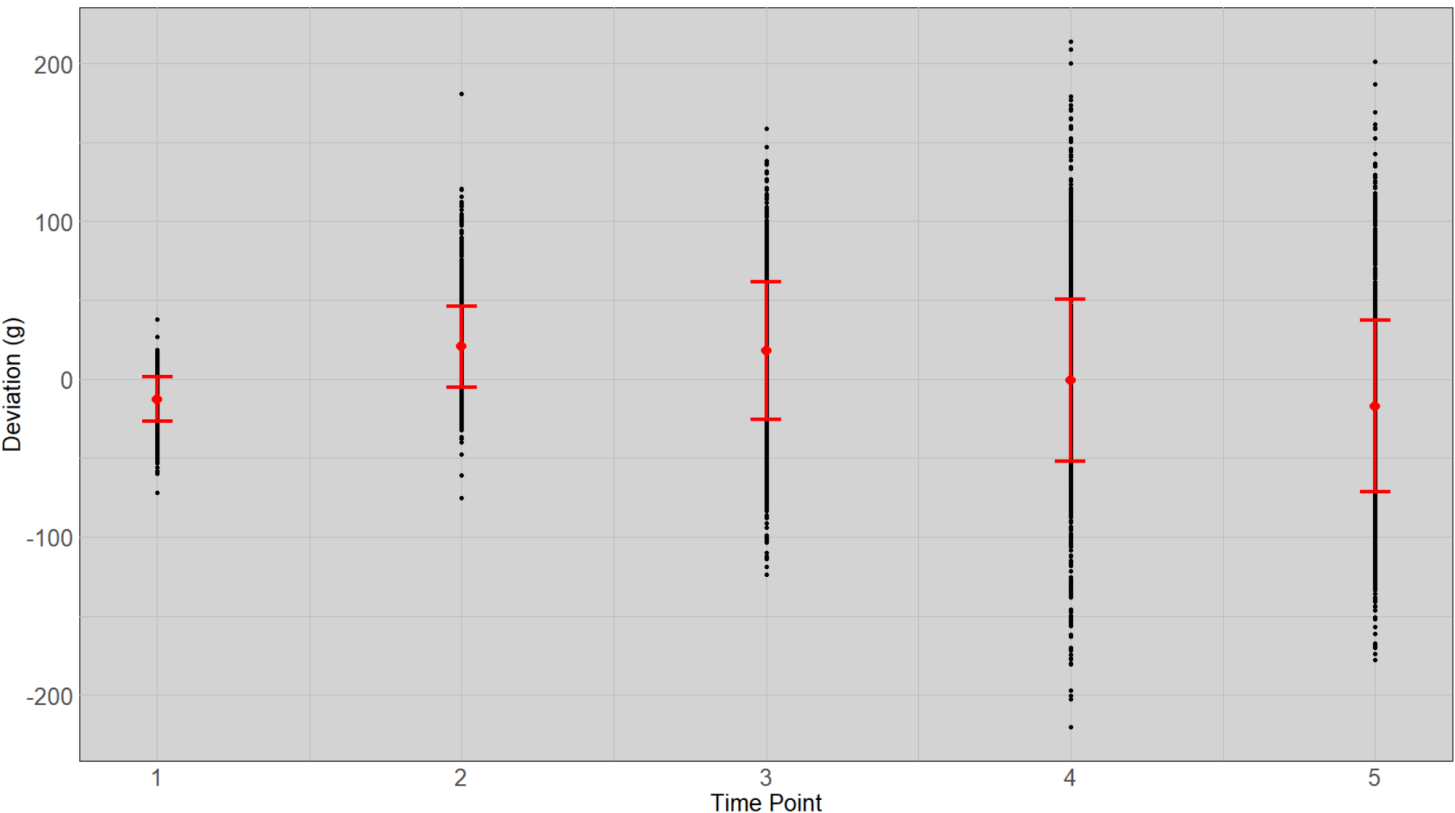
Mean and standard deviation of the observed weight deviation from expected individual weight (gram) with non-linear regression in observed $\:f$ scale at stocking ($\:{T}_{1}$), three interval time points ($\:{T}_{2-4}$: 55, 104 and 167 days) and at harvest ($\:{T}_{5}$: 217 days) for all fish. The weight of each fish is plotted as a dot with the standard error limits shown by two short horizontal lines and the mean is located at the mid-point between these.

**Fig. 3:**
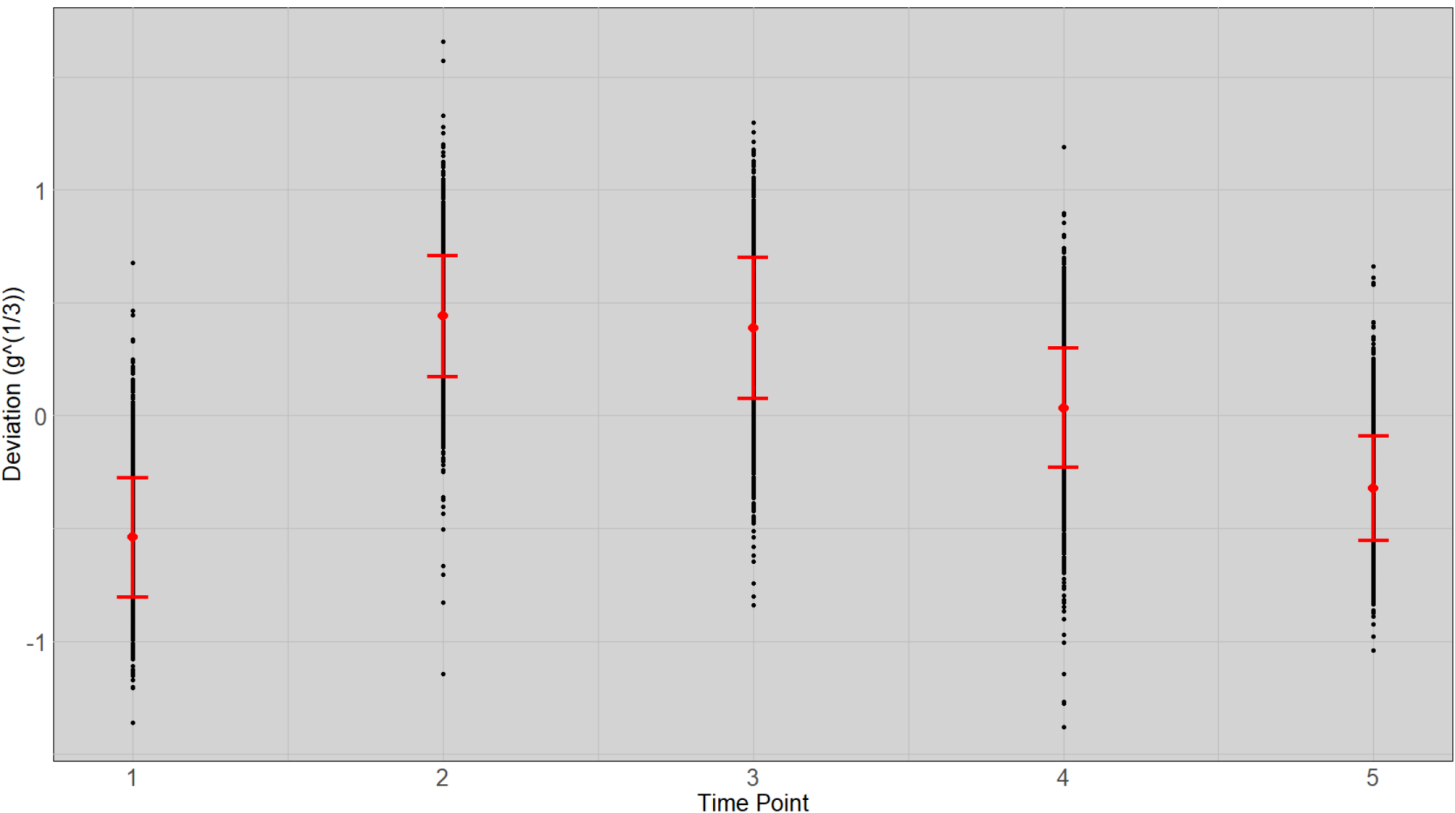
Mean and standard deviation of the standardized weight deviation from expected individual weight (gram) with cubic root transformations at stocking ($\:{T}_{1}$), three interval time points ($\:{T}_{2-4}$: 55, 104 and 167 days) and at harvest ($\:{T}_{5}$: 217 days) for all fish. The weight of each fish is plotted as a dot with the standard error limits shown by two short horizontal lines and the mean is located at the mid-point between these.

We estimated the weight exponent ($$\:f$$) to be 1.77 for the fish in this experiment. The non-linear regression coefficient ($$\:{b}_{i}$$) obtained from Eq. 1, that is equivalent to the daily growth coefficient ($$\:DGC$$) per fish showed heterogeneous variances in expected weight between time points (Supplement 1.). The estimate of the expected weight, obtained using the slope of the linear regression at the $$\:\raisebox{1ex}{$1$}\!\left/\:\!\raisebox{-1ex}{$f$}\right.$$ scale and the deviations from straight-line regression on the $$\:\raisebox{1ex}{$1$}\!\left/\:\!\raisebox{-1ex}{$f$}\right.$$ scale (calculated with Eq. 3; Fig. 1) reduces the heterogeneous variances of the deviations compared to the deviations at the observed scale (calculated with Eq. 4; Fig. 2) and reduces the bias of deviations, particularly at the initial and final time point of measurement, compared to the deviations at the $$\:\raisebox{1ex}{$1$}\!\left/\:\!\raisebox{-1ex}{$3$}\right.$$ scale (calculated with Eq. 5; Fig. 3).

### Descriptive statistics

Descriptive statistics for $\:{LnVar}_{ind}$, $\:{LnVar}_{coh}$, $\:DGC$ and $\:{W}_{5}$ are shown in Table 1. There were no significant differences for the mean $\:{LnVar}_{coh}$ between aerated and non-aerated ponds. Mean $\:{W}_{5}$, $\:DGC$ and $\:{LnVar}_{ind}$ in the aerated pond were significantly higher compared to non-aerated pond (*P* < 0.01). The coefficient of variation for $\:{LnVar}_{ind}$ in the non-aerated pond was higher compared to that for the aerated pond.

**Table 1:** Descriptive statistics of harvest weight ($\:{W}_{5}$), daily growth coefficient ($\:DGC$) and log transformed variance from individual and cohort approach ($\:{LnVar}_{ind}$, $\:{LnVar}_{coh}$).

	Aerated (*N* = 884)				Non-aerated (*N* = 800)			
	mean (sd)	min	max	CV	mean (sd)	min	max	CV
$\:{W}_{5}$ (gram)*	781.04 (265.58)	185.7	1588.6	0.34	579.87 (154.60)	135.5	1003.4	0.26
$\:DGC$ (g^1/1.77^ /day)*	0.16 (0.04)	0.05	0.26	0.25	0.13 (0.03)	0.05	0.20	0.23
$\:{LnVar}_{ind}$*	1.33 (1.04)	-3.33	3.47	0.78	1.03 (1.26)	-3.90	3.53	1.22
$\:{LnVar}_{coh}$	-1.09 (0.98)	-5.68	1.35	0.90	-1.23 (1.03)	-5.27	2.18	0.83

N = number of observations (*n*) in the aerated and non-aerated ponds.

* *t-*tests indicated that mean values were significantly different between the aerated and non-aerated pond (*P* < 0.001).

## Genetic and phenotypic parameters

Table 2 shows the estimated genetic parameters of $\:{LnVar}_{ind}$, $\:{LnVar}_{coh}$, $\:DGC$ and $\:{W}_{5}$ from the aerated and non-aerated ponds. For $\:DGC$ and $\:{W}_{5}$, the heritabilities were higher in the aerated pond than in the non-aerated pond, although the means were overlapped by the standard error limits of the other value. In the non-aerated pond, genetic variances for $\:{LnVar}_{ind}$ were more than four times higher than for $\:{LnVar}_{coh}$, and heritability estimates two times higher. In the aerated pond the estimates were higher for $\:{LnVar}_{coh}$ than for $\:{LnVar}_{ind}$. Heritability estimates for both $\:{LnVar}_{ind}$ and $\:{LnVar}_{coh}$ were higher in the non-aerated pond (0.28 and 0.12 respectively) compared to the aerated pond (0.06 and 0.10 respectively; Table 3).

**Table 2:** Genetic variances (*σ*^2^_A_), phenotypic variances (*σ*^2^_P_), heritability (*h*^2^) of log transformed variance from cohort and individual approach ($\:{LnVar}_{coh}$ and $\:{LnVar}_{ind}$), daily growth coefficient from five weight records $\:DGC$) and harvest weight ($\:{W}_{5}$) and their standard errors (*se*) in the aerated and non-aerated pond.

Trait	Aerated			Non-aerated		
	σ^2^_A_	σ^2^_*P*_	h^2^ ± se	σ^2^_A_	σ^2^_*P*_	h^2^ ± se
$\:{LnVar}_{ind}$	0.060	1.057	0.06 ± 0.03	0.440	1.563	0.28 ± 0.06
$\:{LnVar}_{coh}$	0.091	0.907	0.10 ± 0.05	0.118	0.988	0.12 ± 0.05
$\:DGC$ (g^1/1.77^ /day)	0.25 × 10^− 03^	0.88 × 10^− 03^	0.29 ± 0.06	0.12 × 10^− 03^	0.48 × 10^− 03^	0.25 ± 0.07
$\:{W}_{5}$ (g)	8444.79	37,274	0.23 ± 0.06	2791.11	15,148	0.18 ± 0.06

### Genetic correlations

The genetic correlations between $\:DGC$, $\:{W}_{5}$, $\:{LnVar}_{ind}$ and $\:{LnVar}_{coh}\:$in the aerated pond are shown in Table 3. In the aerated pond, the genetic correlation between $\:{LnVar}_{ind}$ and $\:{LnVar}_{coh}\:$was moderate (0.46). We found moderate and positive genetic correlations between $\:{LnVar}_{coh}$ and both $\:DGC$ and $\:{W}_{5}$ in the aerated pond (0.43 and 0.35, respectively). In contrast, the genetic correlations between $\:{LnVar}_{ind}$ and $\:DGC$, as well as between $\:{LnVar}_{ind}$ and $\:{W}_{5}$ were moderate and negative (-0.44 and − 0.45, respectively).

**Table 3:** Estimated genetic (above diagonal) and phenotypic (below diagonal) correlations of log transformed variance from individual and cohort approach ($\:{LnVar}_{ind}$ and $\:{LnVar}_{coh}$), daily growth coefficient ($\:DGC$) and harvest weight ($\:{W}_{5}$) of tilapia in the aerated pond. Standard errors are in the brackets.

	$\:{\boldsymbol{L}\boldsymbol{n}\boldsymbol{V}\boldsymbol{a}\boldsymbol{r}}_{\boldsymbol{i}\boldsymbol{n}\boldsymbol{d}}$	$\:{\boldsymbol{L}\boldsymbol{n}\boldsymbol{V}\boldsymbol{a}\boldsymbol{r}}_{\boldsymbol{c}\boldsymbol{o}\boldsymbol{h}}$	$\:\boldsymbol{D}\boldsymbol{G}\boldsymbol{C}$	$\:{\boldsymbol{W}}_{5}$
$\:{LnVar}_{ind}$	X	0.46 (0.32)	-0.44 (0.23)	-0.45 (0.24)
$\:{LnVar}_{coh}$	0.32 (0.03)	X	0.43 (0.24)	0.35 (0.26)
$\:DGC$	-0.29 (0.03)	0.07 (0.04)	X	0.99 (0.00)
$\:{W}_{5}$	-0.23 (0.03)	0.10 (0.04)	0.92 (0.01)	X

Table 4 shows the genetic correlations between $\:DGC$, $\:{W}_{5}$, $\:{LnVar}_{ind}$ and $\:{LnVar}_{coh}\:$in the non-aerated pond. In the non-aerated pond, the genetic correlation between $\:{LnVar}_{ind}$ and $\:{LnVar}_{coh}\:$was observed to be low (0.20). We estimated low genetic correlations between $\:{LnVar}_{coh}$ and both $\:DGC$ and $\:{W}_{5}$ in the non-aerated pond, (-0.06 and 0.01, respectively). The genetic correlations between $\:{LnVar}_{ind}$ and $\:DGC$ and those between $\:{LnVar}_{ind}$ and $\:{W}_{5}$ were moderate and negative (-0.68 and − 0.52, respectively).

**Table 4:** Estimated genetic (above diagonal) and phenotypic (below diagonal) correlations of log transformed variance from individual and cohort approach ($\:{LnVar}_{ind}$ and $\:{LnVar}_{coh}$), daily growth coefficient ($\:DGC$) and harvest weight ($\:{W}_{5}$) of tilapia in the non-aerated pond. Standard errors are in the brackets.

	$\:{\boldsymbol{L}\boldsymbol{n}\boldsymbol{V}\boldsymbol{a}\boldsymbol{r}}_{\boldsymbol{i}\boldsymbol{n}\boldsymbol{d}}$	$\:{\boldsymbol{L}\boldsymbol{n}\boldsymbol{V}\boldsymbol{a}\boldsymbol{r}}_{\boldsymbol{c}\boldsymbol{o}\boldsymbol{h}}$	$\:\boldsymbol{D}\boldsymbol{G}\boldsymbol{C}$	$\:{\boldsymbol{W}}_{5}$
$\:{LnVar}_{ind}$	X	0.20 (0.24)	-0.68 (0.12)	-0.52 (0.17)
$\:{LnVar}_{coh}$	0.03 (0.04)	X	-0.06 (0.26)	0.01 (0.29)
$\:DGC$	-0.42 (0.03)	-0.01 (0.04)	X	0.99 (0.00)
$\:{W}_{5}$	-0.33 (0.03)	0.04 (0.04)	0.96 (0.00)	X

**Fig. 4:**
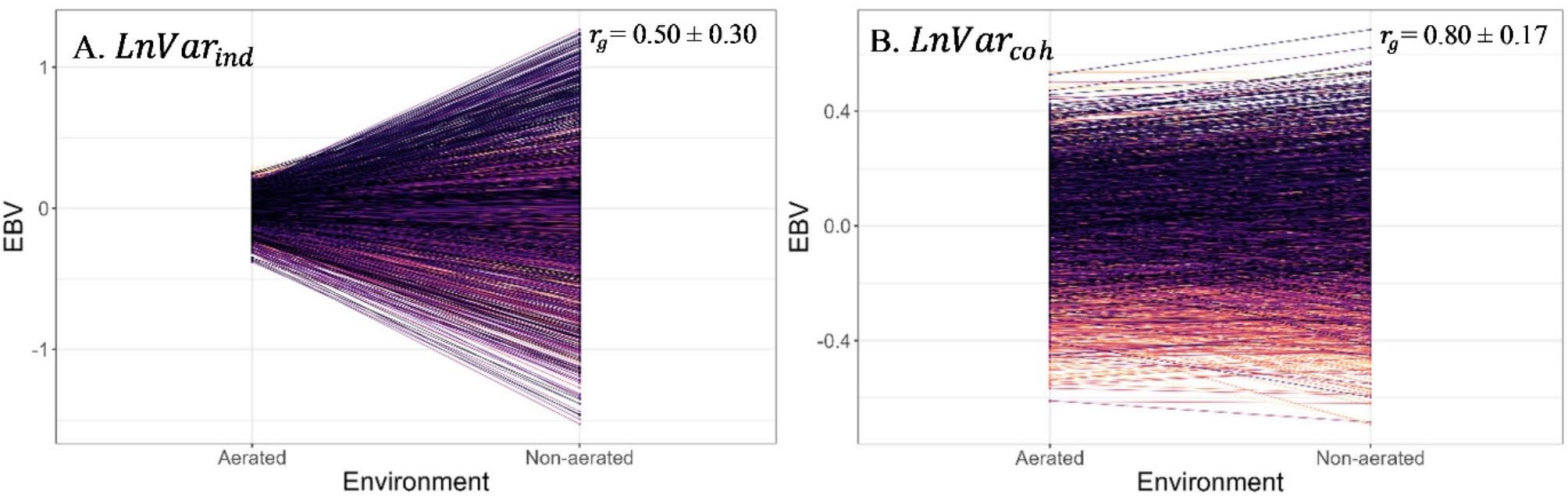
The individual estimated breeding values (EBV) for log transformed variance of deviations from (**A**) individual approach ($\:{LnVar}_{ind}$) and (**B**) cohort approach ($\:{LnVar}_{coh}$) between the aerated and non-aerated pond. The genetic correlations ($\:{r}_{g}$) with standard error are included inside the plot.

The genetic correlations between the aerated and non-aerated pond for $\:{LnVar}_{ind}$ and $\:{LnVar}_{coh}$ were estimated from the bivariate model. The genetic variation for $\:{LnVar}_{ind}$ in the non-aerated pond was higher than in the aerated pond whereas the genetic variance for $\:{LnVar}_{coh}$ in the non-aerated pond was comparable to the aerated pond (Table 2; Fig. 4). The genetic correlation between the aerated and non-aerated pond was lower for $\:{LnVar}_{ind}$ (0.50 ± 0.30) than for $\:{LnVar}_{coh}$ (0.80 ± 0.17, Mengistu et al., 2022). Taking into account the large standard errors, this result shows that $\:{LnVar}_{ind}$ is genetically different in both environments with a substantial degree of genotype by environment interaction (GxE).

## Discussion

This study investigated the resilience indicator $\:LnVar$ when calculated using the individual growth curve as the expected growth performance ($\:{LnVar}_{ind}$). We aimed to improve the definition of $\:LnVar$ to better capture the response of individual fish to environmental disturbances. In the next part, we discuss the comparison of resilience measured with $\:{LnVar}_{ind}$ and $\:{LnVar}_{coh}$, the implications of including $\:{LnVar}_{ind}$ in tilapia breeding programs and the potential for further improvements to calculate $\:{LnVar}_{ind}$ as the indicator for resilience.

### Definition of $\:{\boldsymbol{L}\boldsymbol{n}\boldsymbol{V}\boldsymbol{a}\boldsymbol{r}}_{\boldsymbol{i}\boldsymbol{n}\boldsymbol{d}}$

We calculated $$\:LnVar$$ based on deviations from expected individual weights fitted from weight observations at five time points. To obtain the expected individual weights, we estimated the weight exponent from nonlinear regression of observed weight on the five fish ages. The nonlinear growth model uses regression parameters to describe the shape of the generated curve^15^. Our estimated growth exponent (*f*) of 1.77 is comparable to the growth exponent from studies by Mayer, et al.^19^ and Janssen, et al.^20^ who reported weight exponents for gilthead seabream of 1.54 and 1.63, respectively. The change in fish size between time points typically leads to the heteroscedasticity. However, the calculation of variance of the deviation from straight-line regression on the $$\:\raisebox{1ex}{$1$}\!\left/\:\!\raisebox{-1ex}{$f$}\right.$$ scale removes heteroscedasticity. Furthermore, we fitted the weight data at the $$\:\raisebox{1ex}{$1$}\!\left/\:\!\raisebox{-1ex}{$f$}\right.$$ scale to both a linear model (Model 1) and a quadratic model (Model 2) and compared these two models to investigate if the use of a straight-line regression is reasonable. Although the estimated regression coefficients between Models 1 and 2 are significantly different (*P* < 0.05), the slope of the quadratic term in Model 2 is very small (-0.0002), suggesting that the quadratic term may not add much explanatory power to the model.

### Comparison with $\:{\boldsymbol{L}\boldsymbol{n}\boldsymbol{V}\boldsymbol{a}\boldsymbol{r}}_{\boldsymbol{c}\boldsymbol{o}\boldsymbol{h}}$

We found that the heritability estimate for $\:{LnVar}_{ind}$ was more than two times higher than for $\:{LnVar}_{coh}$ in the non-aerated pond (0.28 and 0.12, respectively). The heritability estimate of $\:{LnVar}_{ind}$ was also four times higher in the non-aerated pond, compared to the aerated pond (0.28 vs. 0.06), while the study by Mengistu, et al.^14^ showed no significant difference in the heritability for $\:{LnVar}_{coh}$ between non-aerated and aerated pond (0.12 ± 0.05 and 0.10 ± 0.05, respectively). These significance differences may indicate that $\:{LnVar}_{ind}$ more accurately captures the response of fish to environmental disturbances than $\:{LnVar}_{coh}$. We measured resilience using $\:LnVar$ based on the deviations of the observed weight from the expected weight. These deviations indicated the response of an individual to the environmental change. The expected weight as the baseline to calculate the deviation should be independent from the environmental change. $\:{LnVar}_{coh}$ was calculated based on the deviation from the mean weight of the fish cohort. Changes in the mean weight of the fish cohort depend on environmental conditions between time points that affect all fish in the cohort in the same manner. If the mean weight of the fish cohort changes due to the environmental effect, the response of individual fish is relative to these “group” changes. Therefore, the calculation of $\:{LnVar}_{coh}$ using the deviation from the mean weight of the fish cohort actually estimates the residual response, as the fish cohort response is already embedded within the mean weight of the fish cohort and is not shown in the deviation used to calculate $\:LnVar$.The expected individual growth curve to calculate $\:{LnVar}_{ind}$ is fitted from five individual weight records and therefore, produces a smoother curve compared to the mean cohort weight that exhibits more erratic behaviour between time points. The expected individual growth curve is independent of the change in environmental conditions and able to disentangle the response of fish cohort to the environmental change. Therefore, using the expected individual growth curve to calculate the deviation in calculation of $\:{LnVar}_{ind}$ can better capture the response of fish to environmental disturbances.

### The implication of including $\:{\boldsymbol{L}\boldsymbol{n}\boldsymbol{V}\boldsymbol{a}\boldsymbol{r}}_{\boldsymbol{i}\boldsymbol{n}\boldsymbol{d}}$ in the tilapia breeding program

$\:{LnVar}_{ind}$ is moderately heritable in non-aerated pond, indicating the presence of additive genetic variance for resilience in the challenging environment. The heritability for $\:LnVar$ in our study was higher than that reported for layer chicken (0.10 ± 0.04^10^) and pigs (0.11 ± 0.03^21^). Berghof, et al.^13^ calculated $\:LnVar$ based on the deviation from the mean weight of the cohort in chicken. Gorssen, et al.^21^ used individual body weight records of pigs which were fitted with a Gompertz growth curve. The heritability estimates for $\:{LnVar}_{ind}$ are higher in non-aerated pond than the aerated pond. Non-aerated pond are typical for smallholder tilapia production systems^14^. We hypothesize that fish grown in non-aerated pond face significant challenges due to daily recurrent hypoxia, leading to increased expression of genetic variation in $\:LnVar$.

Growth remains the primary trait of interest in aquaculture production and breeding programs^22,23^. Understanding the genetic correlation between growth and resilience is essential for optimizing breeding programs for both growth and resilience. The expected correlation between growth and $\:LnVar$ can be explained by resource allocation theory, where energy allocation to cope with environment disturbance may divert energy away from growth^24,25^, leading to decreased fish growth. However, in this study, the genetic correlations between $\:{LnVar}_{ind}$ and both $\:DGC$ and $\:{W}_{5}$ were found to be moderately negative in both aerated (-0.44) and non-aerated pond (-0.68) with high standard error. Here, a negative correlation is favourable for simultaneous improvement in $\:LnVar$ and growth, while a positive correlation is unfavourable. Therefore, selecting for growth in the challenging environment can be expected to improve $\:LnVar$. Simultaneous selection for two traits often results in a negative correlation due to the action of pleiotropic genes, which affect both traits in the desired direction by selection and are rapidly brought toward fixation^26^. The Genetically Improved Farmed Tilapia (GIFT) strain tilapia used in this study had already undergone 17–18 generations selection for growth^14^. The observed favourable correlation between growth rate and $\:LnVar$ suggests that long-term selection for growth has led to increase in $\:LnVar$.

We observed a substantial genotype by environment interaction (GxE) for $\:{LnVar}_{ind}$, as indicated by the genetic correlation of 0.50 between the aerated and non-aerated pond. In non-aerated pond, a correlated response is defined as the multiplication of the genetic correlation between aerated and non-aerated pond, the ratio of genetic standard deviations between aerated and non-aerated pond, and the selection response in aerated pond. When the genetic correlation is less than one, the correlated response is smaller than the direct response, assuming that the heritabilities in the two environments are similar^27^. We used data from the experiment by Mengistu et al.^28^ to estimate the genetic correlation of $\:{LnVar}_{ind}$ between aerated and non-aerated ponds. This estimate was based on a genomic relationship matrix (GRM) derived from SNPs obtained through genotyping-by-sequencing (GBS). Mengistu et al.^28^ highlighted several limitations related to convergence problems: (1) limited sample size, (2) missingness and genotyping errors in GBS data, and (3) mass spawning and unbalanced family sizes. Therefore, refinements are needed, including the use of the complete dataset with approximately 1,000 animals per environment for estimating genotype-by-environment (GxE) interactions^29,30^, reducing missingness and genotyping errors in GBS data, genotyping the parents, and equalizing the number of individuals per family. However, Mengistu et al.^28^ also demonstrated that by clustering animals into groups based on high molecular co-ancestry, individuals with weak genomic relationships to all others could be identified.

Given the presence of GxE between the aerated and non-aerated pond for $\:{LnVar}_{ind}$, it is obvious that the genetic improvement in the aerated selection environment will not be fully realized in the non-aerated production environment. If the breeding goal is to increase resilience in non-aerated production environments and selection must be conducted in an aerated nucleus, it is crucial to integrate information of own individual performance in the aerated environment with relative’s records in the non-aerated environment. This integration of information could enhance selection accuracy and the genetic gain for resilience. A study by Mulder and Bijma^31^ showed that incorporating performance data from the production environment in an index significantly increases genetic gain in that environment if GxE is present. However, caution should be taken due to the limitations of this study, which include the use of only one aerated and one non-aerated pond, and the possibility of a pond effect independent of aeration. Mengistu et al.^28^ showed that that aeration positively impacted harvest weight (26% higher in the aerated pond), improved tilapia survival rates, and resulted in a lower feed conversion ratio (FCR) in the aerated pond (1.73) compared to the non-aerated pond (2.31).

#### $\:{\boldsymbol{L}\boldsymbol{n}\boldsymbol{V}\boldsymbol{a}\boldsymbol{r}}_{\boldsymbol{i}\boldsymbol{n}\boldsymbol{d}}$, as the indicator for resilience

$\:LnVar$ measures the constancy of fish growth during the grow-out period. The constancy of fish growth can be an indicator for the fish’s response to the perturbations. As the available energy for growth at a specific moment is limited, coping with stress, including restoring homeostasis, may divert energy away from growth^4,32,33^ and potentially lead to growth fluctuations. Various mechanisms for coping with environment stress, such as reducing feed intake, decreasing food assimilation and increasing energy demand for maintenance processes, modify energy fluxes, all result in decreased energy proportion for growth^4,5,33^. A study by Folkedal, et al.^34^ showed that fish prioritize coping with the stressor through reduced feeding activity. Later, when the favourable conditions are restored, and food is available, fish compensate for the growth by temporarily accelerating somatic growth^35^. Compensatory growth is characterized by an elevated growth rate from enhanced feed intake and efficiency^36^. This feeding response of fish to environmental stressors, with decreasing feed intake and compensating for growth, may lead to growth fluctuation. We hypothesize that more resilient fish can maintain their feed intake during stress period and may grow more constantly and perhaps better survive environmental perturbations. However, there is limited understanding in this area and further study is needed. Selecting more resilient fish could lead to more constant growth, which plays a vital role in optimizing feeding strategies. In aquaculture practice, farmers predict feeding requirements using information on fish biomass based on the average weight of fish from periodic sampling to avoid under or overfeeding^37,38^. Accurately predicting growth is essential for estimating fish feeding requirement^39^. Optimal feeding strategies improve the feed conversion ratio (FCR), which holds considerable economic value^40^ and reduces environmental impact^41^. Gorssen, et al.^21^ recently showed a moderate and positive genetic and phenotypic correlation between $\:LnVar$ and individual biological FCR in pigs (0.33). Further study is needed to estimate the genetic and phenotypic correlation between individual biological FCR and the constancy of growth measured with $\:LnVar$ in tilapia. The assumption that selecting resilient fish could lead to more efficient growth opens the opportunity to harness the economic benefits from the genetic improvement of $\:LnVar$.

#### Further improvement to calculate $\:{\boldsymbol{L}\boldsymbol{n}\boldsymbol{V}\boldsymbol{a}\boldsymbol{r}}_{\boldsymbol{i}\boldsymbol{n}\boldsymbol{d}}$ as indicator for resilience

The effect of environmental stressors on fish metabolism is evident, but finding evidence for effects on growth is often complex^5,33^. Whole-animal changes such as growth represent the tertiary response of fish to perturbations, following hormonal changes and physiological adjustment, which are the primary and secondary physiological responses, respectively^24^. Stressor exposure may affect fish growth via various factors, including feed intake, food absorption and maintenance energy^4,5,33^. $\:LnVar$, as a resilience indicator, measures the constancy of fish growth in response to perturbations. Understanding the biological mechanisms underlying $\:LnVar$ as a resilience indicator is crucial, as well as confirming its relationships with factors that may influence growth, such as feed intake and feed efficiency. Furthermore, improved resilience could lead to enhanced immunity and disease resistance, as these are categorized as a tertiary response to perturbations, similar to growth. Infectious diseases continue to pose a significant challenge affecting aquaculture productions^23,42^. In chickens, Berghof, et al.^13^ estimated a low genetic correlation between $\:LnVar$ for growth and natural antibodies. However, there is limited understanding, and further research is needed to understand the relationships between $\:LnVar$ and resilience indicators.

Less frequent records and longer intervals between measurements may be sufficient for traits like growth, which reacts more slowly to disturbances than traits measuring physiological response. Mengistu, et al.^14^ and our study found genetic variation in $\:LnVar$ for growth with monthly weight measurements. Calculating $\:LnVar$ for growth requires longitudinal records of individual fish weight. In the context of commercial breeding practices, acquiring longitudinal data has been challenging as it is expensive and requires manual handling, which itself can induce stress in fish^43,44^. Manual handling can cause physical disturbance and acute stress, affecting fish behaviour, welfare and growth^45,46^. Therefore, there is a need for low- or non-invasive tools to enable frequent measurements. Automated phenotyping offers a non-invasive solution, making longitudinal measurements per individual fish more effortless and potentially more accurate^37,44^. Automated phenotyping technology has been developed and applied in various aquaculture species, including salmon, catfish, tilapia and seabream^47–51^. The evolving technology of automated phenotyping in fish will significantly facilitate the application of $\:{LnVar}_{ind}$ as the resilience indicator in breeding programs for aquaculture species.

## Conclusion

In conclusion, we improved the calculation of $$\:LnVar$$ to better capture the response of individual fish to environmental disturbances in the fluctuating environment with $$\:{LnVar}_{ind}$$. $$\:{LnVar}_{ind}$$ should be measured on $$\:\raisebox{1ex}{$1$}\!\left/\:\!\raisebox{-1ex}{$f$}\right.$$ scale to avoid heteroscedasticity. The exponent “*f”* should be estimated directly from the data. $$\:{LnVar}_{ind}$$ was found to be highly heritable in the more challenging environment and this can be exploited by selective breeding. The negative correlation between $$\:{LnVar}_{ind}$$ and growth rate implies that selection for growth may also improve $$\:LnVar$$. Whether selection for $$\:LnVar$$ improves resilience and FCR remains to be tested. We recommend measuring $$\:LnVar$$ through repeated weight records and based on the individual expected growth trajectories in fish breeding programs to simultaneously improve resilience and growth.

## Materials and methods

The experiment was conducted in the Aquaculture Extension Centre, Department of Fisheries, Jitra, Kedah State, Malaysia. The source of the experimental fish is the Genetically Improved Farmed Tilapia (GIFT) Breeding Program that is run by WorldFish in Malaysia. The details of family production and grow-out of tilapia were described by Mengistu, et al.^28^. Below, we summarize family production, nursery, and grow-out of tilapia for this study.

### Family production

We produced our experimental fish using the 16th generation of the GIFT strain as selected parents. We maintained the male and female breeders in separate 9 m^2^ hapas (3 m × 3 m) with a mesh size of 1 cm in an earthen pond for two weeks. Mating was done in four hapas (each 30 m^2^) suspended in a 500 m^2^ earthen pond. Eighteen males and 50 female breeders were stocked in each of the mating cages. In total, 72 males and 200 females were used. We conducted this mating process for 15 days. On the sixteenth day, the parents were removed, and the fry were kept in the same cages for a nursing period of 60 days.

### Grow-out period

The fingerlings from each net cage were transferred into one of four aerated tanks after the 60 days nursing period and conditioned for three days before tagging. A random sample of fingerlings was anesthetized using clove oil and individually tagged using PIT (passive integrated transponder) tags. At tagging, a 1 cm^2^ fin clip sample was collected and PIT tag number and body weight were recorded. Equal numbers of individually tagged fingerlings from each nursery cage were randomly allocated to two earthen ponds. In total, 1570 fish were stocked in each pond with a stocking density of 3 fish/m^2^. The size of each of the pond was 511 m^2^ with a water depth of 1 to 1.2 m. To test the effect of oxygen availability on resilience in growth, we created two different environments: One of the ponds was aerated using a paddle wheel and blower to create a normoxic environment. The second pond was without aerators which resulted in natural diurnal dissolved oxygen (DO) fluctuations.

### Trait measurements

#### Longitudinal measurement

We measured the weight of tilapia at five time-points: at stocking ($\:{W}_{1}$: day 1), three interval time points ($\:{W}_{2-4}$: 55, 104 and 167 days) and at harvest ($\:{W}_{5}$: 217 days). Fish from the non-aerated and the aerated pond were always measured on two consecutive days.

The calculation of $\:LnVar$ needs the expected performance from which the observed deviation can be calculated. In this study we calculated $\:LnVar$ from the individual expected growth trajectories ($\:{LnVar}_{ind}$). We compared $\:{LnVar}_{ind}$ to the calculation of $\:LnVar$ as used by^14^ (hereafter cohort approach: $\:{LnVar}_{coh}$). The methods differ in their approach to calculate the expected performance.

#### Calculation of $\:{LnVar}_{ind}$

$\:{LnVar}_{ind}$ was calculated from the deviations of observed weights from the expected weights of the individual at timepoints $\:{W}_{1}$ to $\:{W}_{5}$. To obtain the expected weights we fitted an exponential curve to the observed weights:

1 $$\:{W}_{it}=a+{b}_{i}*{t}^{f}$$

 

where $\:{W}_{it}$ is the weight of fish *i* at age $\:t$, *a* is the intercept, $\:{b}_{i}$ is the slope of the non-linear regression for fish *i*, $\:t$ is the fish age and $\:f$ is the overall weight exponent. The growth curve exponent $\:f$ was estimated for the fish in this experiment using the *nls* function in R^52^. The non-linear regression coefficient ($\:{b}_{i}$) obtained from Eq. (1), is equivalent to the daily growth coefficient ($\:DGC$) per fish. Then, we transformed the five observed weights per fish as $\:{W}^{\frac{1}{f}}$ to linearize the growth curve and we estimated $\:DGC$ per fish as the slope of the linear regression of $\:{W}^{\frac{1}{f}}$ on the age of the fish at the five time points *t* and calculated the expected weight of individual fish at times *t*:

2 $$\:{W}_{exp,it}^{\frac{1}{f}}=\:a+{DGC}_{i}*t$$

where $\:{W}_{exp,\:it}$ is the expected weight of fish *i* at age $\:t$, *a* is the intercept, $\:{DGC}_{i}$ is the daily growth coefficient as the slope of the non-linear regression for fish *i*, $\:t$ is the fish age. Per fish, we then calculated the deviations ($\:{dev}_{it}$) as:

3 $$\:{dev}_{it}=\:{W}_{obs\:it}^{\frac{1}{f}}-\:{W}_{exp\:it}^{\frac{1}{f}}$$

Where $\:{dev}_{it}$ is the deviation of observed weight from expected weight of fish *i* at time point *t*, $\:{W}_{obs\:it}$ is the observed body weight of fish *i* at time point $\:t$ and $\:{W}_{exp\:it}$ is the expected body weight of fish *i* at time point $\:t$. Next, for each fish, we calculated variance of the resulting five deviations (*Var-dev*) and transformed the *Var-dev* using the natural logarithm to obtain $\:{LnVar}_{ind}$.

To investigate the effect of the heteroscedasticity for the deviations between time points, we compared the deviations that were calculated at $$\:\raisebox{1ex}{$1$}\!\left/\:\!\raisebox{-1ex}{$f$}\right.$$ scale in Eq. (3) with the deviations that were calculated at $$\:f$$ scale (observed) and at $$\:\raisebox{1ex}{$1$}\!\left/\:\!\raisebox{-1ex}{$3$}\right.$$ scale (Eqs. 4 and 5, respectively). The growth exponent 3 is commonly used for fitting growth curves of fish with rounded shapes that grow in volume^15,53–55^. The deviations ($$\:{dev}_{t}$$) from $$\:f$$ scale were calculated as:

4 $$\:{dev}_{t}=\:{W}_{obs\:t}-{W}_{exp\:t}$$

Then, the deviations ($\:{dev}_{t}$) from cubic scale were calculated as:

5 $$\:{dev}_{t}=\:{W}_{obs\:t}^{\frac{1}{3}}\:-\:{W}_{exp\:t}^{\frac{1}{3}}$$

Where $\:{W}_{obs\:t}$ is the observed body weight at $\:t$ and $\:{W}_{exp\:t}$ is the expected body weight at $\:t$.

#### Calculation of $\:{LnVar}_{coh}$

The expected performance for $\:{LnVar}_{coh}$ is defined as the mean weight of fish that belong to the same cohort^14^. Fish cohort is defined as the fish belonging to the same nursery hapa, sex and grow-out pond. The details calculation for $\:{LnVar}_{coh}$ were described by Mengistu, et al.^14^. We refer to $\:LnVar$ based on expected weights from the cohort as $\:{LnVar}_{coh}$.

### Genetic parameter Estimation

Records from 1686 genotyped fish were available for genetic analyses. Genomic relationship matrix was computed based on 11,293 SNPs using the calc_grm program^56^ with the vanraden2 option, as described in Mengistu, et al.^14^. Phenotypic and genetic variances of $\:{LnVar}_{ind}$, $\:{LnVar}_{coh}$, $\:DGC$ and harvest weight ($\:{W}_{5}$) were estimated using ASReml version 4.2 ^57^ fitting a bivariate animal model with a genomic relationship matrix. Phenotypic ($\:{r}_{p}$) and genetic ($\:{r}_{g}$) correlations between the four traits within the aerated pond and within the non-aerated pond were estimated from bivariate linear models. We used the following animal model:

6 $$\:{y}_{ijk}=\mu\:+{\mathrm{C}\mathrm{A}\mathrm{G}\mathrm{E}}_{i}+{\mathrm{S}\mathrm{E}\mathrm{X}\:}_{j}+{\mathrm{b}\mathrm{*}\mathrm{S}\mathrm{W}}_{k}+\:{\mathrm{a}}_{k}+{\mathrm{e}}_{ijk}$$

where: $\:{y}_{ijk}$ is the vector of $\:{LnVar}_{ind}$, $\:{LnVar}_{coh}$ $\:DGC$ and $\:{W}_{5}$ for the univariate models or two of those traits for the bivariate models; $\:\mu\:$ is overall mean; $\:{\mathrm{C}\mathrm{A}\mathrm{G}\mathrm{E}}_{i}$ is fixed effect that accounts for nursery hapa effects (*i* = 1– 4); $\:{\mathrm{S}\mathrm{E}\mathrm{X}}_{j}$ is the fixed effect of sex (*j* = male, female, unknown); $\:{\mathrm{S}\mathrm{W}}_{k}$ is a covariate start-weight of the *k-*th individual (included only for estimation of harvest weight: $\:{W}_{5}$); $\:{\mathrm{a}}_{k}$ is random additive genetic effect of the *k-*th individual; $\:{\mathrm{e}}_{ijk}$ is the random residual effect associated with an individual. We calculated the heritability as the ratio between additive genetic variance ($\:{\sigma\:}_{A}^{2}$) and phenotypic variance ($\:{\sigma\:}_{P}^{2}$), $\:\frac{{\sigma\:}_{A}^{2}}{{\sigma\:}_{P}^{2}}$.

Genetic and residual correlations between traits in the same environment were obtained from bivariate analysis. The animal effects for bivariate model were distributed as N(0,G⊗C) with the additive genetic variance covariance matrix (C) is $$\:\left[\begin{array}{cc}{\sigma\:}_{A,1}^{2}&\:{r}_{A,12}{\sigma\:}_{A,1}{\sigma\:}_{A,2}\\\:{r}_{A,12}{\sigma\:}_{A,1}{\sigma\:}_{A,2}&\:{\sigma\:}_{A,2}^{2}\end{array}\right]$$, and G is the genomic relationship matrix, $\:{\sigma\:}_{A,1}^{2}$ and $\:{\sigma\:}_{A,2}^{2}$ is the additive genetic variance of trait 1 and trait 2. $\:{r}_{A,12}{\sigma\:}_{A,1}{\sigma\:}_{A,2}$ is the additive genetic covariance between trait 1 and trait 2. The residuals were distributed as N(0, I⊗R) with residual variance-covariance matrix (R) is $$\:\left[\begin{array}{cc}{\sigma\:}_{e,1}^{2}&\:{r}_{e,12}{\sigma\:}_{e,1}{\sigma\:}_{e,2}\\\:{r}_{e,12}{\sigma\:}_{e,1}{\sigma\:}_{e,2}&\:{\sigma\:}_{e,2}^{2}\end{array}\right]$$, where I is an identity matrix, $\:{\sigma\:}_{e,1}^{2}$ ($\:{\sigma\:}_{e,2}^{2}$) is the residual variance of trait 1 (trait 2), and $\:{r}_{e,12}{\sigma\:}_{e,1}{\sigma\:}_{e,2}$ is the residual covariance between trait 1 and trait 2. Genetic and phenotypic correlations among traits were calculated as the covariance divided by the product of the standard deviations of the two traits.

We estimated the genetic correlation between the same traits measured on different (related) individuals in the aerated and non-aerated ponds with the bivariate model 1. The additive genetic variance-covariance matrix is the same as the bivariate model 1 where $\:{\sigma\:}_{A,1}^{2}$ is the additive genetic variance for the traits in the aerated pond, $\:{\sigma\:}_{A,2}^{2}$ is the additive genetic variance for the traits in the non-aerated pond and $\:{r}_{A,12}{\sigma\:}_{A,1}{\sigma\:}_{A,2}$ is the additive genetic correlation between aerated and non-aerated ponds.

The covariances of residuals between environments was set to zero, as a fish performed in only one environment. The residual variance-covariance matrix is $$\:\left[\begin{array}{cc}{\sigma\:}_{e,a}^{2}&\:0\\\:0&\:{\sigma\:}_{e,na}^{2}\end{array}\right]$$ where $\:{\sigma\:}_{e,a}^{2}$ is the residual variance for the trait in the aerated pond and $\:{\sigma\:}_{e,na}^{2}$ is the residual variance for the trait in the non-aerated pond.

## Data Availability

The data that has been used is confidential and can be obtained by requesting WorldFish or Animal Breeding and Genomics, Wageningen University & Research (John W.M. Bastiaansen; email: john.bastiaansen@wur.nl).
